# Preparing for and Executing a Randomised Controlled Trial of Podoconiosis Treatment in Northern Ethiopia: The Utility of Rapid Ethical Assessment

**DOI:** 10.1371/journal.pntd.0004531

**Published:** 2016-03-11

**Authors:** Henok Negussie, Thomas Addissie, Adamu Addissie, Gail Davey

**Affiliations:** 1 Brighton & Sussex Medical School, University of Sussex, Falmer, Brighton, United Kingdom; 2 School of Social Anthropology, Addis Ababa University, Addis Ababa, Ethiopia; 3 School of Public Health, Addis Ababa University, Addis Ababa, Ethiopia; Centers for Disease Control and Prevention, UNITED STATES

## Abstract

**Background:**

Community-based randomized controlled trials are often complex pieces of research with significant challenges around the approach to the community, information provision, and decision-making, all of which are fundamental to the informed consent process. We conducted a rapid ethical assessment to guide the preparation for and conduct of a randomized controlled trial of podoconiosis treatment in northern Ethiopia.

**Methods:**

A qualitative study was carried out in Aneded *woreda*, East Gojjam Zone, Amhara Regional State from August to September, 2013. A total of 14 In-depth Interviews (IDIs) with researchers, experts, and leaders, and 8 Focus Group Discussions (FGDs) involving 80 participants (people of both gender, with and without podoconiosis), were conducted. Interviews were carried out in Amharic. Data analysis was started alongside collection. Final data analysis used a thematic approach based on themes identified *a priori* and those that emerged during the analysis.

**Results:**

Respondents made a range of specific suggestions, including that sensitisation meetings were called by *woreda* or *kebele* leaders or the police; that Health Extension Workers were asked to accompany the research team to patients’ houses; that detailed trial information was explained by someone with deep local knowledge; that analogies from agriculture and local social organisations be used to explain randomisation; that participants in the ‘delayed’ intervention arm be given small incentives to continue in the trial; and that key community members be asked to quell rumours arising in the course of the trial.

**Conclusion:**

Many of these recommendations were incorporated into the preparatory phases of the trial, or were used during the course of the trial itself. This demonstrates the utility of rapid ethical assessment preceding a complex piece of research in a relatively research-naive setting.

## Author Summary

Community-based randomised controlled trials in developing countries, especially in settings with no research experience, are faced with significant challenges around approaching the community and obtaining informed consent. We conducted a Rapid Ethical Assessment, a simple and quick qualitative technique involving interviews and focus group discussions with researchers, experts, community leaders, and community members of both gender, with and without podoconiosis, the condition being studied. We aimed to explore optimal methods to provide information about the trial and approaches to the consent process preferred by the community. Overall, suggestions were grouped into five domains: approaching patients, information provision and comprehension, decision making, constraints to participation and ways of explaining randomisation and the control group. These recommendations were used to design the information sheet and consent form and to make other changes before and during the trial. This study highlights the utility of rapid ethical assessment prior to clinical trials involving complex procedures and concepts.

## Introduction

The Informed Consent (IC) process is a mandatory requirement in clinical research, and its relevance and specifications are clearly outlined in international, legal and ethical guidelines such as the Nuremberg Code[1], The Declaration of Helsinki[2], The Belmont Report[3] and The Council for International Organizations of Medical Sciences (CIOMS)[4]. IC describes a process in which an individual voluntarily agrees to participate in a research study after the purpose, risks and alternatives have thoroughly been described. The ethical principle of Respect for Persons identifies the consent process as containing three key elements: the provision of information, comprehension of information, and voluntary participation[3]. Researchers are required to provide potential participants with sufficient information about a trial. This should include the study objectives, design and procedures to be followed, identification of procedures which are experimental, risks and benefits of participation, expected duration of the study, their rights as participants, a disclosure of alternative courses of treatment, extent of confidentiality of records, and an explanation of whom to contact for questions about the research and research subjects’ rights. Potential participants must be allowed adequate time to make a voluntary and informed decision about participation. Provision of information in a language prospective participants are capable of understanding is critical in obtaining genuine informed consent before they are enrolled in a trial[4–5].

Comprehension of information provided about the research influences the decision to participate in a scientific investigation. Many factors shape approaches to implementing informed consent, including the nature and cultural context of the study[6]. Studies have shown that information provided in information sheets and informed consent documents may be completely alien to potential participants. For example, differences between research and free treatment or aid and randomisation may be difficult for participants to understand[7]. In low-income countries, low literacy levels, limited access to health care, lack of familiarity with clinical research and decision-making and consent procedures, may all affect understanding [6, 8]. Thus, when clinical trials are to be conducted in low-income countries, especially in communities with little or no previous experience of participating in research, providing information that is both comprehensive and comprehensible may be very challenging.

Additionally, although consent is normally documented in a written, signed and dated consent form, written documentation of informed consent for studies can be particularly problematic in certain cultures[9]. In some areas of the world, both individuals and communities may be reluctant to put their signatures or thumbprints on a document because of previous experiences of having signed “legal” forms that resulted in political, economic and social victimisation including loss of property and sanctions against them [10].

Both these issues demonstrate the need to orient requirements specified in international guidelines to the local context in which a study is to be conducted. Relevant training for fieldworkers, discussion with stakeholders, communicating with participants at different stages and providing incentives are important for successful recruitment and retention of subjects in clinical trials[11]. A genetic study conducted in southern Ethiopia recommended that researchers should carefully assess tradition of decision making in communities. For example, in the context of southern Ethiopia, families should be approached before individual participants[12].

Rapid ethical assessment (REA) is a brief qualitative intervention designed to map the ethical terrain of a research setting prior to recruitment of participants and has been proposed as one means of improving the consent process in research-naïve settings [13–14]. It uses rapid ethnographic techniques to better understand the ethical issues that are relevant to a specific piece of research in a given study setting. The approach has been used prior to research projects in Ethiopia, Cameroon and The Gambia, and has been shown to be feasible in the Ethiopian context [15].

Podoconiosis is a form of lymphoedema (leg swelling) arising in people going barefoot in highland tropical areas[16]. A recent nationwide mapping indicated clusters of high prevalence (> 5%) were exclusively found in Amhara, Oromia, and Southern Nations Nationalities and People’s Regional State, which represent most of the central highlands of Ethiopia[17]. Despite the high impact of podoconiosis on rural farming communities, treatment has been hampered by misdiagnosis (chiefly confusion with filarial lymphoedema) and fatalism[18]. We conducted a REA to guide the preparation for and conduct of a randomized controlled trial of podoconiosis treatment in northern Ethiopia. The trial is being conducted in East Gojjam Zone, Amhara regional state in northern Ethiopia. The primary object of the trial is to test the hypothesis that community-based treatment of podoconiosis lymphoedema reduces the frequency of acute dermatolymphangioadenitis episodes (ADLA, ‘acute attacks’) and improves other clinical, social and economic outcomes. In this study we aimed to explore optimal methods of conveying information about the trial and the approaches to obtaining informed consent preferred by the community in which the trial was to be carried out [19].

## Methods

### Study Site

The study was carried out in Amber *woreda* (district), part of East Gojjam Zone in Amhara regional state, where the prevalence of podoconiosis in the adult population is 3.4% [20]. The *woreda* is located 280 km North West of Addis Ababa and 20 km south east of Debre Markos, the zonal capital. According to the 2007 Census, Aneded *woreda* is made up of 19 *kebeles* (smallest administrative unit) and has a total population of 91,224, with 89,446 living in rural *kebeles* [21]. It has 4 Health Centres and 20 Health Post (HPs). Each HP is staffed with two trained Health Extension Workers (HEWs), female civil servants implementing the health extension package at *kebele* level under the direct supervision of Health Centers and the *Woreda* Health Office[22].

According to the Ethiopian Demographic & Health Survey 2011, 62% of women and 37% of men in Amhara Region cannot read at all[23].

### Study design

In-Depth Interviews (IDIs) and Focus Group Discussions (FGDs) were the main methods of data collection. Items in the semi-structured interview and focus group guides used were adapted from rapid ethical assessment instruments previously used in Ethiopia and the Gambia [9, 13]. Slightly differing interview schedules were used for each category of respondent. Items in the guides were presented in the same order as anticipated in the trial, i.e. approaching patients, provision of information, decision making, constraints and randomisation. In addition, the interview guide for researchers and experts included items about how comprehension of information given about the study should be assessed and application of international ethical guidelines in the local context. The study was approved by the Research Governance and Ethics Committee of Brighton & Sussex Medical School and the Institutional Review Board of the College of Health Sciences, Addis Ababa University. Introductory letters were presented to regional, zonal and *woreda* level health authorities.

### Participants

These included researchers from Debre Markos University who had conducted studies in the area, podoconiosis prevention and treatment service providers from International Orthodox Christian Charities (IOCC), *woreda* level health and agricultural experts, development agents and health professionals, *kebele*, community, *iddir* (a form of social insurance group) and religious leaders. Community participants were identified by Health Extension Workers (HEWs) and a group of ten participants took part in each FGD from three *kebeles* based on their distances from the *woreda* capital: remote and less remote. Accordingly, *Wonga Nifasam*, *Yewobi* and *Mislawash kebeles* were included. A total of 14 IDIs and 8 FGDs were planned. Separate FGDs for people with podoconiosis and non podoconiosis-affected community members of both sexes were conducted.

### Data collection

The REA was conducted from July to August 2013. Data were collected by a public health expert and a social anthropologist (both Ethiopians). Individual participants were informed about the objectives of the study and oral consent obtained. Once permission to participate was secured, interviews were recorded using digital recorders. The interviews were conducted in offices and community settings. After the first few interviews, data collection activities were halted until previously collected data were analyzed. This was to identify themes that emerged and modify interview guides accordingly. Interviews and FGDs were conducted in the local language (Amharic), and on average lasted one to one-and-a-half hours, respectively. Core questions for IDIs and FGDs are given in Table 1.

**Table 1:** Core questions for in-depth interviews and focus group discussions.

Theme	Sample question
Approaching patients	From your experience, who in the community do you think can serve as the most practical way of gaining access to and approaching prospective participants for the trial?
Provision and comprehension of information	In your opinion, what fundamental information about the trial would you say should be provided to prospective participants? From your experience, how does the community prefer to receive information about trial? **Additional items for researchers and experts**: How do requirements in international ethical guidelines compare with local circumstances in Ethiopia? In your opinion what do you think are the challenges for the practical implementation of requirements in international ethical guidelines in the Ethiopian context? How do you suggest patients’ understanding of information given about the study be assessed?
Decision making	From your experience how would you explain the norm of decision-making about important situations such as participating in a study in this community? What roles do heads of families, family members, relatives and women have in making decisions? How much time would you say prospective participants need to reach a decision?
Constraints to participation	In your opinion, what factors influence patients’ decision to participate in the study?
Explaining randomisation and delayed intervention	From your experience what local narratives exist to explain the concept of randomisation? Can you think of techniques to promote understanding of why some will receive treatment immediately and some at a later date? How can the benefit of participating in the trial be explained? (E.g. for delayed treatment group?)

### Data analysis

Data analysis was started alongside collection. First, data were transcribed and translated into English. This was followed by categorizing and organizing data thematically in accordance with the objectives of the study. Final data analysis was made using a thematic approach based on themes identified *a priori* and those that emerged during the analysis.

## Results

A total of 94 participants (42 females and 52 males) took part in 14 in-depth interviews and 8 Focus Group Discussions (FGDs—10 participants each). The age of participants ranged between 19 and 80 years. The majority, 79, were married, while 5 were not married and 10 were widowed. The qualifications of the health professionals and experts ranged from diploma to Master’s degree level. Almost all community participants had little or no formal education (Table 2). In the following paragraphs, study findings are organized under the following thematic areas: approaching patients, provision and comprehension of information, decision making, constraints to participation and explaining randomisation and delayed intervention.

**Table 2:** Characteristics of the study participants.

Characteristics	Number
Sex	
Male	52
Female	42
Average age (in years)	38.5
Marital Status	
Married	79
Single	5
Widowed	10
Educational Status	
Can read and write	10
Cannot read and write	70
Elementary and High School	5
Higher education	9
Podoconiosis status	
Affected	40
Non-affected	54

### Approaching patients

The way researchers approach communities and individual participants to provide information about a study plays a vital role in obtaining informed consent. The approaches described here could be seen as being at two levels: first approaching the community as a whole followed by individual participants. Some suggested *kebele* leaders and law enforcement as key to approaching the community and individual participants

“The *kebele* leader will guide you…he knows all the patients. The local police department know where all the patients live, and the two can work together…they’ll call patients to a meeting and everyone will attend, since they respect and obey the orders of the *kebele* leader” (P5, Non-patient, Male).

“Without the *kebele’s* assistance, it will be like walking in darkness…you can’t go without the *kebele* helping you!” (P6, Non-patient, Male).

Regarding the order of approach, community members suggested that *kebele* officials should be approached first. These would then arrange a meeting with the community either in a *kebele* compound or in a church on a specific religious day, and information about the study could be provided.

“For example, you can use St. Michael’s day when everyone will be attending the Church (P7, Patient, Male).

Once the community is informed about the intended study, researchers can proceed to approach individual patients for consent. Participants indicated that individuals should be approached for consent at their homes in one of two ways. The first would be to identify and train locally known individuals and send them to patients’ houses. Secondly, researchers could go to patients’ houses accompanied by a local guide.

“We take them [people coming from other places] to peoples’ houses in the community, greet them and explain the purpose of our visit, they’ll listen and give their responses, we then continue to next household” (P7, Non-patient, Female).

A range of views was expressed on researchers going house-to-house unaccompanied. Focus group participants explained that patients would not refuse to meet anyone offering help to treat their illness:

“It is for their own benefit, so they don’t need a lot of explanation, they’ll accept an invitation to participate” (P3, Non-patient, Female).

However, most preferred the assistance of the Health Extension Workers (HEWs) in reaching individual patients.

“The responsibility should only be given to HEWs, as they go house-to-house to deliver the various health service packages. Thus, Health Extension Workers know where there’re pregnant women, if children were absent from school and also houses of podoconiosis patients” (Podoconiosis expert 1).

### Provision and comprehension of information

In this section, the responses included are mainly those of researchers and podoconiosis experts because focus group participants rarely discussed the kind of information that should be provided.

International ethical guidelines require that information about potential risks, inconvenience or restrictions should be balanced against any possible benefits. It is very interesting and rather worrying to note, however, that the expert participants in this REA repeatedly suggested de-emphasizing information on risks or possible negative consequences that participation in the study may have on individuals.

“We stress the purpose of the study and its importance in improving patients’ conditions…patients should not feel too much about risks” (Podoconiosis expert 1).

“When informed about risks, although some may rarely occur, people focus more on risks instead of the benefits…your study has minimal risks associated with participation, except maybe the discomfort of being identified as a podo patient” (Podoconiosis expert 3).

This invited a follow up question about applicability of other aspects of international ethical guidelines locally. Participants believed one size would not fit all and that international guidelines needed to be contextualized.

“The main problem is adopting guidelines prepared by western standards if we provide levels of information suitable for literate people to those who’re illiterate; it will become boring and unclear for most community members” (Podoconiosis expert 3).

Similarly, although international ethical guidelines require provision of relevant information such as contact details of researchers and their institutional affiliation, to assist participants with information needs and to answer queries, reflecting on previous experience, a researcher said:

“I don’t think the majority in our community will phone requesting additional information…we have never given contact information to individual participants [laughing]…that information exists only on paper [study document/proposal]…if and when they ask, mostly we show them the introductory letter from the *woreda*” (Researcher 2).

Different views were offered regarding whether information should be provided in groups or individually -

“It is preferable to provide information as a group, for example, for people gathered in a *kebele* compound…once information about the study is presented, inviting a discussion will help to get people’s opinions. The majority are illiterate so using flyers would be difficult” (Expert 1).

“We approached individuals on a house-to-house visit…explained the purpose of the study, asked willingness to take part, and where agreed, asked for signature, as indication of agreement. Then Health Extension Workers collected the data” (Researcher 1).

When it comes to who should provide information, some thought that anyone could provide information, while others thought the person providing information should thoroughly understand the cultural milieu of the area -

“Someone who is from that community understands the culture and is trusted by the people” (Researcher 2).

In relation to checking comprehension of information, the simplest way would be asking potential participants to recount the most important aspects of information provided -

“To check the extent of understanding, ask a few questions focusing on important elements of the study, repeating questions if necessary” (Researcher 1).

A few FGD participants maintained there was no need for information, and that requesting willingness to participate suffices, because in the end it is for their own benefit:

“First of all no one will refuse to participate, since they’re all ill” (P3, Patient, Female).

“We all want to be healthy and thus no one will refuse” (P4, Patient, Female).

Finally, when it comes to signing the written consent form, participants stressed the importance of understanding the study. Experts who had experience conducting research in the area said:

“I’ve seen people agreeing orally, but refusing to sign the consent form. This is because they fear the purpose is to confiscate their lands…you’ve to clearly explain the purpose and meaning of signing the form…if they trust you, they’ll sign” (Podoconiosis expert 1).

“People in our communities are very fearful of signing anything. If asked to sign something, they always think it is linked with some form of legal issues, something that will later result in a legal obligation and responsibility. But, can be achieved through discussion, especially if community leaders are involved in discussions and made to understand the purpose clearly” (Podoconiosis expert 2).

### Decision making

Participants suggested that consent to participate depends on the type of research. For example, for short projects and those involving interviews and surveys, obtaining consent may not be that difficult. However, for long term studies such as the trial in question, which demand considerable investment of time from participants, the decision may require permission of other family members, especially if the potential participant happens to be the family breadwinner. Others thought there was no general formula for reaching decisions -

“It varies according to the situation…parents decide on behalf of children. Heads of households both men and women, decide for themselves” (Podoconiosis expert 3).

Several others said that the decision to participate rested with individual patients:

“It is the patients themselves who make that decision” (Community leader).

“They shoulder the disease burden and its social consequences…patients decide for themselves” (Researcher 2).

Others thought that the authority to make a decision differed for men and women. Men could decide for themselves while women needed to consult and get their spouse’s approval before consenting

“What we commonly see is, women don’t decide without first consulting with and obtaining their husband’s permission” (Podoconiosis expert 2).

However, female participants asserted that both men and women decide after discussing with family members. Where women are household heads, (through divorce or widowhood), they decide without consulting others -

“We decide after discussing with family members and considering their advice…but sometimes we can decide ourselves” (P2, Non-patient, Female).

“Since we ask men for advice, they do the same before they decide” (P6, Non-patient, Female).

“I discuss with my neighbours, I don’t decide alone” (P3, Patient, Female).

“I decide on my own, I don’t have anyone to discuss with” (P5, Patient, Female).

Some suggested that in the context of rural Ethiopia, any important decision had to involve significant other people; family members, relatives, neighbours, community elders and religious leaders -

“It is hard to get consent without any influence from others…in this area [Amhara region]; community leaders have a big influence” (Podoconiosis expert 3).

“In rural areas, individuals don’t live distinct from others…individuals’ decisions will be affected by others and vice versa. Therefore, for long term studies, it’s appropriate to obtain the family’s permission too” (Researcher 1).

### Constraints to participation

Although focus group participants all agreed there were no clear reasons hindering patients from participating in the trial, in-depth interviewees argued reasons that might deter individuals and explained ways to deal with them -

“I think people will be willing to participate, but my concern is our community has expectations, such as monetary rewards and sometimes for time they spent, when asked, especially by people from other areas to participate in some activity” (Health Professional).

Participants also said false rumours about the study’s purpose may create misconceptions which will impact on decisions to participate. Fertile ground for rumours may be created by botched community sensitisation, and misleading information reaching communities before the study team had a chance to discuss directly with individuals. In order to prevent misconceptions, garnering the support of community, religious and political leaders, ensuring clear understanding of the nature of the study at all levels and proceeding to enter the community in as short a time interval as possible after district level sensitisation are all important -

“Rural communities are small, close-knit villages, and word about any outside visitors will spread very quickly…personal beliefs such as ‘visitors are talking to people with swollen legs and their families, it looks like they’re going to give them aid’ will start to fly around” (Podoconiosis expert 1).

“First, authorities at different levels need to clearly understand the purpose of the study, risks and benefits followed immediately by entry into the community to break any rumours that may arise in the intervening period” (Podoconiosis Expert 3).

“People with no connection with the study may be the ones that start false information…if non-affected people know the purpose of the study; they can stop rumours when they arise (*Woreda* Health Office expert).

### Explaining randomisation and delayed intervention

The main object of this REA was to furnish information on how best to design the consent process for a randomised controlled trial of podoconiosis treatment, so conveying the concept of randomisation was expected to be very important. In the following paragraphs, understanding of randomisation and local analogies used to help explain randomisation is presented. This is followed by suggestions on how to keep participants in the delayed treatment group motivated to continue participating in the trial even when not receiving treatment.

Several local analogies exist that could be used to explain randomisation. The following social organisations were mentioned -

“Everything from ‘*Iqqub’*, ‘*Mahiber*’ and ‘*Senbete*’ work based on the concept of chance…it is important to show people that everyone has been registered before the lottery is drawn; simply tell people, we’ll draw similar to *iqqub* and those who win will be treated immediately and the rest will wait for a year…they’ll understand” (Podoconiosis expert 1, and Health Professional).

[‘*Iqqub’* is an Amharic term used to describe a traditional saving system where people form groups and pay an agreed amount of money at an agreed interval into a common pool. Each member of the group, selected randomly, will receive one large sum of money, and this continues until everyone has received the sum they’ve contributed in the end. ‘*Mahiber*’ and ‘*Senbete*’ are terms referring to a voluntary and mutual religious gathering of a group of people in a church or at home to celebrate a common religious (saint’s) holiday while feeding the poor, on a rotational basis.]

A health professional with considerable experience working in the *woreda* provided two examples of explaining randomisation through agricultural analogies used previously by agricultural experts assessing poultry farming and the use of modern fertilisers in the community:

“Most people were suspicious of modern fertilizers, but after a comparison of the use of compost with modern fertilizer was made, farmers witnessed the difference in the year’s crop both yielded and began to use modern fertilizers. A similar example could be the chicken farms…there was a problem of which breed to use; foreign or hybrid chickens, then experts demonstrated the difference in the number of eggs laid and convinced farmers” (*Woreda* Health Office expert 1).

Several other analogies for randomisation were given in patient and non-patient focus groups. “*Kircha”* is an Amharic term to describe the traditional system for sharing the meat of slaughtered animals (usually a cow or an ox) and “*Worefa*” a description of waiting in line or of a waiting list:

“Yes, we know and use lottery method for ‘*kircha’*…but, we haven’t seen this in meetings” (P All, Non-patient, Female).

“Lottery is lottery and ‘*worefa*’ is ‘*worefa*’, they’ll understand it (P1, Non-patient, Male).

“Most of the things we do, we do them by ‘*worefa*’ even we wait in line to be blessed by the priest’s cross…even you put firewood one after the other, not all at once (P4, Non-patient, Male).

“When we take out the herd for grazing, we cast lots to decide who will tend to the cattle first, second and so on” (P6, Non-patient, Male).

Participants suggested several ways to explain delayed treatment

“Explain you are testing the effectiveness of the treatment, if after 12 months’ of observation the treatment needs modification, we will modify it and the delayed group will get the improved treatment…they will understand, most have lived with the disease for a long time so waiting for another year will not be intolerable” (*Woreda* Health Office expert 2).

“Assure them that they will get treated in a year and that probably they will get a better, improved treatment, next year” (Researcher).

“These people have lived with podoconiosis for long and do not mind waiting for another year. It is also important to inform them that although this is a small scale intervention, the findings will be expanded to a large number of patients in other parts of the country” (Podoconiosis expert 2).

“You should have coffee ceremonies with patients to discuss and inform them the number of patients assigned in this group who are also waiting. Also explain that the result will be of benefit not only for them, but for a lot of other patients in other places” (*Woreda* Health Office expert 1).

Participants in the FGDs said:

“My sister is a podoconiosis patient and this is how I’ll explain it to her…it’s based on chance, if her chance allows, she’ll get treated now otherwise she’ll wait for some time and get treated” (P6, Non-patient, Male).

“Everyone will participate and no one will feel unhappy to wait…it’s like walking on a narrow road where people travel in a row; one in front and another following, in the end both will reach their destinations…the second group will get treated later and won’t mind waiting”(P6, Non- patient, Male).

“Yes, we’ll wait, it is based on chance and lottery is not biased, does not favour anyone” (P2, Patient, Female).

“Tell them in a way everyone can understand…for example, when the government builds roads, they can’t build all the roads at the same time, it takes time…similarly, in this research, those who get the chance will get treated first and others have to wait” (P3, Patient, Male).

Several views were put forward regarding encouraging continued participation of the delayed treatment group. These ranged from explaining the waiting list scenario at the IOCC treatment and prevention project, mentioning the average waiting time, to regular meetings with the control group, to discussing over coffee to explain the wider benefits. Several respondents felt it was important to guarantee treatment for the control group after a year. However, others seemed to suggest since all are ill and desperate, none would complain, or drop out.

“You need to be able to provide an incentive for the group that has to wait for a year. I don’t think they will wait for a year without getting treated” (Researcher 1).

“More focus should be given to those in the delayed treatment group, as they may be expected to complain. First, explain that a year is a short period to wait; some have waited for 7 to 10 years to get treatment. Then explain that random selection will be used to assign people into one of the groups, they’ll understand and be contented no one was favoured” (Podoconiosis expert 1).

In sum, although there seemed to be a range of approaches, most participants appeared confident that participants would stay in the study given these explanations.

## Discussion

This Rapid Ethical Assessment (REA) provided useful insights into many aspects of preparing for and conducting a randomised controlled trial of podoconiosis treatment in northern Ethiopia. The term REA originated from Rapid Ethnographic Assessment techniques and is a preliminary investigation of potential ethical issues in a setting prior to launching a study. However, it is to be noted here that the word ‘rapid’ in Rapid Ethical Assessment, should not be interpreted as hastening the ethical review process. In addition, REA is not a *sine qua non* for all scientific research. As ethical issues in research are a function of the subject of inquiry, design of the study, population being studied, the setting in which the study takes place and availability of resource, these and other factors determine the need for the conduct of REA. Accordingly, researchers in the field suggested this determination be reviewed in the light of specific type of research proposed and provided a possible list of working criteria[15]. For example, studies in research-naïve communities, dealing with a sensitive issues and vulnerable population, clinical trials including studies involving collection of biologic specimen often require REA[24].

The 6 weeks’ fieldwork activities for this REA cost around 31, 000 ETB (US $1,550). Both team members had qualitative research skills and the anthropologist had previous REA experience. A recent assessment on feasibility of REA in three community based studies in Ethiopia estimated an average of 42,815.5 ETB (US $2,140) and duration 4–6 weeks to conduct an REA[15].

Specific strategies adopted by the trial team included holding sensitisation meetings called by *woreda* or *kebele* leaders (though not the police); asking Health Extension Workers to accompany the research team to patients’ houses; employing a local health education expert to provide information about the trial; using analogies from agriculture and local social organisations to explain randomisation; allowing time for individuals to discuss participation with their families before committing to the trial; offering small packets of coffee to participants in the ‘delayed’ intervention arm as an incentive to continue in the trial; and using key community members to quell rumours that arose in the course of the trial. We discuss these strategies in the context of participants’ responses below.

Respondents suggested that information about the study should be provided through initial sensitisation meetings called by local administrative leaders or the police. Health Extension Workers who are known to and trusted by the community could then be asked to guide trial team members to patients’ homes. A similar approach was used in southern Ethiopia, where sensitisation meetings preceded introductions to specific patients, although in that context, members of the local Patient Association accompanied the research team to patients’ homes rather than Health Extension Workers [13]. The approach adopted for this trial was first to give information to *woreda* representatives, *kebele* chairs, community and religious leaders, who all disseminated the information further. Health Extension Workers were then briefed and asked to list patients with podoconiosis and lead trial team members to their houses.

Using the police to approach communities for sensitisation about a study has not been reported in other contexts, and we were surprised this group was mentioned. However, a recent case study among police officers, academics, civil society and the judicial sectors in Amhara regional state, suggests that community policing has improved community-police relations in the region. The manner in which community members view Community Police Officers has gradually changed so they are now considered to be respected community representatives who play a range of social service functions that fall outside traditional policing purviews [25]. However, this approach was not employed, given that its potential for undue influence and the impact it might have on the voluntariness of the informed consent process was unclear. Whether these roles might be extended to community sensitisation would be an interesting focus for future research.

Participants preferred information about the trial to be delivered by a person who understands the local culture. While the entire trial team spoke Amharic (the language used in this setting), and several had grown up in Amhara region, we found that local variations in dialect meant that detailed information was best conveyed by a health educator from East Gojjam zone itself. This individual joined the trial team for enrolment days, explained the trial, answered questions and probed comprehension before consent was requested [26]. In previous studies which have employed REA, consent processes have significantly improved in terms of both comprehension and decision making as a result of the knowledge and understanding gained [15] as well as facilitating ethically and scientifically sound practice around recruitment and retention of trial participants[13].

In the trial, information was initially given to patients as a group where they were encouraged to ask questions. Their understanding of this information was verified when consent to participate was requested individually. This mirrored procedures followed after REA in Wolaita, southern Ethiopia, where in the subsequent genetic study, information was provided through group discussions and decisions were reached after consultation with family members, neighbours and friends[9]. The information given in the present trial included the objectives of the study, study procedures, risks and benefits of participation, participants’ rights, how information was to be stored, confidentiality of personal record, and who the investigators were. This was despite many experts in this REA suggesting playing down the risks of participation. They justified concealing risks on the assumption that potential participants would find complete disclosure ‘boring’ or incomprehensible. A similar study in southern Ethiopia reported that the information participants most wanted to hear was the expected benefit of the research[9].

Underestimation of the potential risks attached to research, particularly in the context of a clinical trial, would weaken the informed consent process [2–4, 27]. Informed consent presupposes that subjects understand the potential risks and benefits of their participation, and that it is research, not therapy, in which they will participate [28]. Misunderstood study risks have critical ethical consequences. For example, without fully understanding a study’s risks, participants cannot make an informed decision as to the level of risks they are willing to accept by participating. The Declaration of Helsinki specifies that adequate communication of study risks is imperative for ethical research[2]. Accordingly, in the four-page information sheet the following relevant information was explained (Box 1). In addition, all study staff including investigators and fieldworkers were required to take an online International Conference on Harmonisation, Good Clinical Practice (ICH-GCP) course and certification (Globalhealthtrials.org).

**Box 1: Sections of the trial Information Sheet and Consent Form**

**Heading**

Study title: Randomized controlled trial of podoconiosis treatment in northern Ethiopia

Lay title: A study of the treatment of podoconiosis in northern Ethiopia

Names and institutional affiliations of investigators

**Main body**

Introduction; Your illness; What is IOCC; What is the study about; Study procedures; How are participants selected; How are participants allocated to study arms; What will participating involve; Are there risks or disadvantages to me of participating in the study; Are there any benefits to me of participating in the study; What happens if I refuse to participate; Where can I get alternative treatment; Who will have access to my information; What happens to the blood sample; What if I have any questions*; Who allowed this research to take place; Who is organizing and funding the research; Your understanding of the information we gave you about the study and Consent Form.

*Footer

Names, institutions and contact telephone numbers for further enquiries (if needed):

About the GoLBet trial: the trial co-ordinator and data manager; Head of *Woreda* Health Office; Amhara Regional Health Bureau

About participants’ rights and trial related safety: The trial’s Local Safety Monitor; Institutional Review Board, College of Health Sciences, Addis Ababa University.

Some participants identified differential decision-making power between men and women. Participants in women’s focus groups argued that the decision should be consultative within the family, for both men and women. In addition, they said that where women were heads of households through divorce or widowhood, they could decide without consulting others. While most participants in a REA conducted in Cameroon advocated male responsibility for giving consent[29], it appears that in the context of northern Ethiopia, women can be approached for and provide consent in consultation with their families or by themselves. Unlike the REA study in Cameroon[29], but in line with the one in southern Ethiopia[9], the permission of local traditional authorities and community leaders was not required before individual consent to participate in a study was given. The role suggested here for local community and religious leaders, was to facilitate entry into the community and rectify false information about the study when it arises. Proxy decision-making is not advocated in this community, but has been by other REA respondents, for example in north-west Cameroon (21). National and international guidelines for ethical conduct in research recognize that some standards, such as that requiring individual informed consent be given voluntarily by competent participants, must be met whatever the cultural context within which research is conducted [3–4, 27, 30].

Although participants in focus groups could not identify factors that might hinder participation in the trial, experts raised some concerns. For example, the longer the gap between *woreda/kebele* level sensitisation and approaching individual patients, the more likely that misleading information would reach the communities. In order to prevent misconceptions, garnering the support of community, religious and political leaders to quell rumours was thought to be important. The trial team used this approach during enrolment in one *kebele*; to counter misconceptions being spread by individuals who were opposed to the trial. For example, during group information provision at enrolment, prospective participants questioned whether the treatment works at all based on information from a fellow patient (who refused participation) claiming health professional advice that this is an incurable disease and that the only treatment to improve the condition is surgical removal of the affected leg. In another instance, subjects speculated that the true intent of the trial, as rumour had it, was religious conversion.

Although some participants considered randomisation to be the most difficult concept for prospective participants to understand, several strategies were suggested. Agricultural analogies were thought to be useful ways of explaining the two intervention groups. The concept of the lottery method (‘*Ita’* in the Amharic language) is well known in these communities and is used in social, financial and religious activities. These analogies were all used at times in explaining the trial to potential participants.

To encourage continued participation of the control group, respondents suggested that the IOCC waiting list was used as an example of delayed treatment. Other suggestions included guaranteeing treatment for the control group through meetings at different stages of the trial, and providing small incentives to the control group in the form of packets of coffee to encourage continued participation in the trial. All these strategies were used in the actual trial. For example, the IOCC waiting list scenario and waiting time was explained in the information sheet. In addition, at regular follow-up study visits, participants in the delayed treatment group were informed of the time remaining before they received treatment and given small packets of coffee. It was also explained that participation would help provide evidence on the effectiveness of the treatment for policy formulation at national and international levels, and thus benefit a large number of people with podoconiosis in other places.

There are several limitations of this study. Despite efforts to identify the most productive sample to answer the study questions, by varying location, participants of the focus groups were selected by Health Extension Workers, which may have introduced selection bias. This in turn may have resulted in social desirability bias. For example, HEWs might have selected people they know or who they thought or trusted to provide the “right” responses rather than a “troublemaker”. Focus group participants seemed to suggest there was no reason that patients might decline to participate. This might be because they themselves thought the project was treatment instead of research.

In order to reduce the effect of the ‘therapeutic misconception’ in the actual trial, the difference between research and treatment was clearly explained in the information sheet. For example, the section of the information sheet about the study was explained as follows: “…we’re trying to learn more about the effectiveness of the treatment of podoconiosis. Experience from NGOs so far has shown the current treatment for podoconiosis to work and to be safe. However, more research is required to test the effectiveness the treatment. Unlike projects which are mainly focused on provision of prevention and treatment services, the GoLBet [the trial] study is mainly focused on finding out evidence about the effectiveness of the current treatment and make it widely available in the future for everybody’s benefit”. In addition, randomisation and delayed treatment were explained (Box 2).

**Box 2: Section of the Information Sheet explaining randomisation and delayed treatment**

**How are patients allocated to study arms?**

Assume the *woreda* agriculture office wanted to check the effectiveness of a new fertilizer in terms of yield and making the soil fertile before giving the fertilizer to all farmers in the *woreda*. The office selected plots from farmers who volunteer to take part, spread the fertilizer only on some selected plots and not on the others. Then, a year later they compared the yield of plots with the new fertilizer with those without it. Based on this comparison, the agriculture office would either provide the fertilizer to all farmers or would not. Similarly, the GoLBet will test the effectiveness of the treatment of podoconiosis on patients who are willing to take part in the study by assigning them into one of two study groups: ‘immediate’ treatment’, or ‘delayed treatment’. Those in the ‘immediate’ treatment group will receive treatment immediately for a year while those in the ‘delayed' treatment group will be offered the same treatment a year later. This will help us find out if there is any difference between the two groups by closely watching the progress of everyone in this study. A total of 680 patients (340 in each arm) will participate in the study with duration of one year participation in the study for any given patient. The decision on which any person gets to be allocated to which group will be decided by a system based on chance, not by anyone in the research team. Study numbers we give for individual patients who meet the selection criteria will be fed into a computer which will make allocation based a lottery method. This will be done by experts at Kilifi Clinical Trials Facility in Kenya.

Finally, during the provision of the information and before individual consent was given, subjects were encouraged to ask questions and describe in their own words important aspects of the study including the aims and procedures of the study, awareness of risks and benefits, privacy, voluntary participation, awareness of the possibility of withdrawing at any time and understanding how to get further information.

Interviews were conducted in Amharic, and analysis was done in English. Preferably, qualitative data analysis should be conducted in the language of interview to minimise loss of meaning during translation.

In closing, this REA provided useful information for preparing for and conducting a randomised controlled trial of podoconiosis treatment in northern Ethiopia. This study highlights the utility of rapid ethical assessment prior to clinical trials involving complex procedures and concepts.
